# In-depth analysis of primary schools performance, financial strength and challenges in Bench-Sheko, Kafa and Sheka Zones, Ethiopia

**DOI:** 10.1016/j.heliyon.2022.e09116

**Published:** 2022-03-16

**Authors:** Miressa Yadessa, Matheas Shemelis

**Affiliations:** Mizan_Tepi University, Ethiopia

**Keywords:** Ethiopia, Ethiopian schools, Gross enrolment rate, Gender parity index, Dropout rate, Repetition rate

## Abstract

This study is aimed to look at the performance and challenges of public primary schools found in Bench-Sheko, Kafa, and Sheka Zones. National standards developed for Education Sector Development Program V (ESDP V, 2016–2020) was used to measure the schools’ performance. Primary and secondary data are used to meet the research target. Performance reports of the schools of three consecutive years were thoroughly examined. Public primary schools found in the districts and town administration of the three zones were particularly selected for the purpose of the study. School principals, vice-principals, supervisors, district education bureau officers, and senior teachers were used as the subject of the study. Both quantitative and qualitative information was gathered and analysed. A X^2^-test of independence model is used to see associations between variables. The finding of the study revealed that the schools were progressing well in access. However, there are variations of access rates from district to district and from school to school. The variations were determined by the commitment of the school leadership and the extent of community engagement in school matters. The study further identified the schools as inefficient which is manifested by increasing dropout and repetition rate. Demographic pressure, financial incapability, weak students’ learning interest, poor school infrastructures, poor salary structure, and incentives to keep in and attract talented teachers to the schools were identified as major challenges affecting the performance. The researchers recommend to the policy makers and educational institution leaders to design inclusive policy and financial reform of primary schools with critical implementation and monitoring to come out of these vicious circles.

## 1 Introduction

In many nations of the world general education is taken and implemented as a part of fundamental human right provisions since the endorsement of the Universal Declaration of Human Rights (UDHR, 1948). Particularly, primary education is considered as compulsory to help the development of human personality. For this matter, almost all nations of the world structurally allocate budget, politically support, regularly supervise, evaluate and recommend its implementation. The success of these goals is monitored by core performance indicators like gross enrolment rate, gender parity index, public funding for universal education, and qualified teachers (Peppler et al., 2000). Ethiopia has endorsed relevant international conventions and commitments including the Universal Declaration of Human Rights (UDHR, 1948), Education for All (EFA), and Millennium Development Goals (MDG). The country has used access, efficiency, equity and quality as a major performance indicator of primary education in its Education Sector Development Program (ESDP V,2015/16). However, there is a gap between the target set and the realized performance both at the national and global levels (Global monitoring report, 2015). The goal set to realize universal education for all at the end of 2015 is not comprehended and the ambition to get all children into school is not achieved. In fact, during the recent decades, regions including Ethiopia have demonstrated a considerable change of access to primary education though variations among countries have decreased for consecutive years.

It is apparently true that even today millions of children did not get access to education due to poverty, geographic location, and security. Research findings on the achievement and challenges of Education for All (EFA) have shown that since the campaign conducted on the world education forum at Dakar in 2000, many nations have increased the performance of their education system with recognizable effort. This is manifested by an increase in enrolment ratios and decreased number of students who have never been to school. Besides, Ethiopia has decreased the percentage of children out of school by remarkable rate from 67% in 2000 to 28% in 2011 (Education for All Achievements and Challenges, 2015). On the other hand, it is believed that financing education system and the extent of the progress of education for all is strongly linked. Primary education expenditure according to global monitoring reports of many countries has increased. Though Ethiopia has made significant progress in access to education, there is also a financial challenge faced to meet the target set in Education Sector Development Plan IV (ESDP IV, 2015). For this matter, equitable access to primary education, reduced repetition and dropout rate, improved gender parity index, and improved students’ achievement at primary schools are not fully answered yet. In this case, according to Education Sector Development Program V, (ESDP V, 2016/15-2019/20), despite the dramatic achievement in access, equivalent attainment to higher grades is not apparent. The report stated that many students leave the schools earlier which is reflected in grade 8 completion rate of 47%. The average gross enrolment rate at the beginning of 2016 was 64% of which 63% was for girls and 65% was for boys.

**Graph 1:**
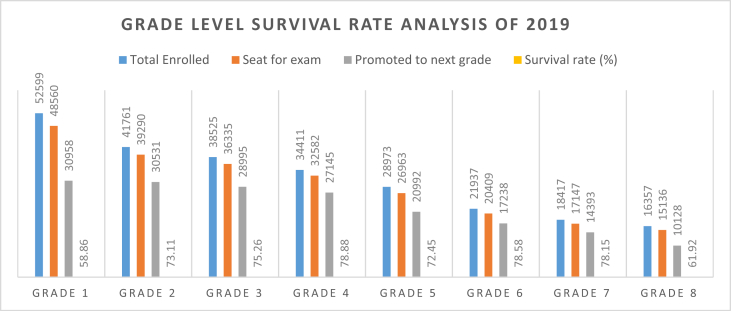
Grade level survival rate analysis of 2019.

However, the target set was 100%. This implies that the target is not achieved. These low enrolment rate at the primary school level indicates the persistent challenges to reduce dropout and repetition. For the fact that targets set in ESDP IV were 1% for dropout and 1% for repetition, performance against these targets, however, was poor. As ESDP V begins repetition rates persist at around 8% and dropout remains at 11%. In addition, the zonal educational statistics abstract shows most primary schools in the study were operating below the expected achievement plan set in the strategy document of sustainable development goal. Bench-Sheko Zone (one of the study sites) annual statistics abstract of primary education of grade 1 up to grade 8’s average survival rate (efficiency) in 2019 was 72.15 % and was planned to reach their primary education efficiency rate at 100% though it is unrealistic. This depicts that it is suffering from primary education wastage. This particularly shows the high dropout and repetition rate recorded starting from grade 1 and became the worst at grade 8. The following Graph 1 shows the analysis of class level inequality of pupils learning progress of study area.

Ethiopia education policy which was formulated in 2002 states that the main purpose of primary schools is to offer basic education to prepare students for further advanced education. The policy set that primary schools shall be exempted from education fees to reduce burdens from parents. It simultaneously indicated that community contribution and involvement in schooling is an important means of financing education. However, in practice, it is difficult to get a complete picture of financial contributions from the communities. As per the (ESDP V, 2015/16), the government of Ethiopia’s expense on education has increased to 70% in a real situation. Of this primary education, spending accounts for 32%. The financial strength and effective utilization of the fund are not clearly known yet. Besides, the ever-increasing number of school-age children and the financial constraints are challenging the government’s ability to finance and provide the needed quality primary education.

Primary schools’ performance is influenced by access, qualifications of teachers, leadership capacities, and representation of female leaders in leadership positions. However, the status of these determinant factors is not known in the study area. With the physical expansion of primary education, financial situation, price of inputs like education materials supplies and efforts to retain teaching staff in the teaching profession by paying good incentives need to be clearly known. For these matters, the researchers believe that the situation of primary schools’ performance needs to be studied for further intervention, clarification, and strengthening. The major factor for low education efficiency which is the major headache of primary schools needs to be examined. This study is aimed to find out the reality regarding the performance (access, efficiency, and equity), financial strength, and challenges of the public primary schools of Bench-Sheko, Kafa, and Sheka zones of Ethiopia. These zones are found in Southern Nations, Nationalities, and People’s Region (SNNPR), South-Western part of Ethiopia. The area is well known for its numerous Ethnic groups, particularly characterized by its multicultural, multilingual, and multi-religious nature of social compositions. The original inhabitants of the area are Kafficho, Bench, Shekicho, Me’en, Suri, Zilmamo, Sheko, Dizi, and Majang peoples. Amharic and other local languages are spoken around the towns. Almost all rural communities are speaking their own native languages and very few of them speak two and more than two local languages. In terms of economic activities, forest resources, and agricultural products are major means of rural communities’ livelihood. Crops mainly coffee, fruits, and spices are the main source of income. As a matter of fact, the responsibility to provide primary education services to the communities is mainly vested in the government. A very small number of private schools (one or two schools) are found in towns of the zones and almost no private schools are found in the rural areas. Other than their limited number, their main target is to collect profit. Children from a family with good economic background have a good chance to join these schools.

## 2 Methods and materials

This part of the paper introduces the readers to the detail of the research design employed, population of the study, sampling techniques, key methods used for data collection, nature and the sources of the data, and model used for data analysis.

This study was descriptive in nature due to the intention of the study was to describe the existing situations regarding the performance of public primary schools; financial capacities and challenges found in Bench-Sheko, Kafa, and Sheka zones. The geographic location of these zones and districts is far away from the center or capital of the country. It is believed that school performance is influenced by access and qualifications of teachers, leadership capabilities, financial strength, and stakeholders’ commitment in which schools near to the center have a more comparative advantage. Though this area is known for its cash crops and spices growing, it is identified by its poor infrastructure developments. A similar situation is observed regarding primary schools found in these districts. In addition to the presence of disparities in performance by location and socio-economic situations, the researchers believed that lack of holistically designed study in the area itself justifies the need to conduct and examine this issue to scientifically identify the problems and tackle the gaps. To realize this target, the study used both primary and secondary data in order to measure the objectives set. Primary data is collected from school principals, cluster supervisors, district education officers, and teachers. The schools’ annual performance report documents are used as secondary data. Documents which was analyzed include mainly the annual educational abstract of each school organized as per their districts. Reports of primary schools organized into 22 districts and 3 administrative towns were reviewed as secondary data. Both qualitative and quantitative approaches for data collection were employed.

Primary data was collected using a structured questionnaire. The survey was distributed to the schools by the enumerator through district education officers. A semi-structured interview was used for some variables. There were focus group discussions held at each district education bureau. The participants for Focus Group Discussion (FGD) was purposively selected based on their school leadership experiences and responsibilities or leadership role held in the schools. Six FGD was organized and conducted by the two researchers and one enumerator. For each FGD conducted one principal, two vice principals, one-unit leader, and four most senior staff have participated. To increase the quality of the data to be gathered through FGD, the interest of the participants and favorable situations were considered based on their consent. In addition, six different district education officers were interviewed on the overall educational achievements and challenges of schools to triangulate whether the performance report of the schools dictates the reality on the ground based on their regular supervision findings. Primary schools’ annual report documents were used as secondary data and mainly included annual educational abstracts and official reports of schools according to their district.

In this study important ethical considerations were made. The researchers have protected the dignity of the respondents. Their freedom to decline participation in the study had maintained for the respondents who filled the questionnaire, attended focus group discussion and interviews. The confidentiality of the research data is safeguarded against violation of their privacy. Specifically, the respondents were communicated about the purpose of the study and freedom to fill the questionnaire in the introduction section. Accordingly, those who received the questionnaire have showed their willingness, properly filled and returned the questionnaire.

Frequency distribution, mean, and chi-square test models were used according to their importance and meaningfulness. Mainly Chi-square test of independence is employed to test whether there is association between primary schools’ cycles (i.e. first cycle is from grade 1 up to grade 4 and second cycle from grade 5 up to grade 8) and changes in education access from year to year; associations between gender and school-age population of different years; gender and gross enrolment rate trends; gender and students’ dropout trends and gender and repetition rate trends across different years. Interview and Focus Group Discussion data was narrated and integrated into the quantitative data analysis by categorizing it into themes.

## 3 Discussions

This portion of the paper provides the details of data analysis and presentation with specific reference to the objectives of the finding. It further discusses the results of the finding regarding primary schools’ performance, financial strength, and challenges based on both qualitative and quantitative output. In specific terms, this part dictates the result of the finding on education access, efficiency, and quality that is manifested by the nature of school-age pupils; gross enrolment rate; net intake rate; dropout and repetition rate; teacher-student ratio; gender parity index (GPI); financial strengths and challenges of primary schools used in the study.

In Ethiopian education system, it is known that primary education life lasts 8 years which itself is divided into a basic education cycle covering grade 1–4 (first cycle) and a general primary cycle covering grade 5–8 (second cycle). It is critical education level to repair the education quality of the nation and basic for later education and economic growth. First cycle primary school is mainly to attain literacy, numeracy and basic awareness about oneself and ones surrounding (environment). The second cycle of primary school and first cycle of secondary school is designed to give graded general education. Primary education is followed by two years of general secondary education (grades 9–10), and two years of preparatory secondary education (grades 11–12). Senior secondary education prepares students for appropriate subjects at tertiary education including vocational and technical education to give training at different levels. Higher education is to produces career-oriented professionals in different fields of study. Education quality, access, equity, and internal efficiency are major standards emphasized as national priorities and measured by indicators. The following table indicates the number of first and second cycle public primary schools of Bench-Sheko, Kafa and Sheka Zones in 2017, 2018 and 2019 academic years.

At the grass-root level, it is observed that increased enrolment of students to the schools has created congestion in the classroom. Table 1 above indicates the number of primary schools of both first and second cycle in the years 2017, 2018, and 2019. The total number of primary schools was increasing from the year 2017 to the year 2019. A _X_^2^ - test of independence model is performed to examine the relation between the cycles and change in access to school at different years. To see whether the difference is statistically significant at each cycle, x^2^-test of independence with α = 0.05 is used. Accordingly, the result shows that the difference observed is not statistically significant at x^2^ = 0.341, df = 2, and p-value = 0.879. An increase in access to primary school implies that the government has played a major role in the expansion of primary education to meet ESDP V targets and providing overall policy guidance and monitoring of the entire sector. This is revealed by the increase in the total number of primary schools built by the government between the 2017 and 2019 academic years which was increased from 1052 schools to 1097 schools and increased government expenditure on education.

**Table 1:** Trends in number of public primary schools of the zones in different years.

School by their Cycles/Academic year	2017	2018	2019
Total number of first cycle primary schools	148	143	149
Total number of second cycle primary schools	904	928	948
**Total**	**1052**	**1071**	**1097**

Conforming with Education statistics annual abstract (2016), government effort to increase the expansion of primary schools is recognizable. This implies that on the one hand the government effort to increase education access opportunities, responding to the socio-cultural problems that females and other children encountered to participate or get opportunities to schooling. In support of this finding, according to the study which was conducted in Tanzania on primary education expansion and quality of schooling, the rapid increase in primary enrolment leads to large increases in the teacher-student ratio and a reduction in teacher quality, (Christine, 2015). Cognizant of this fact it is important to know the effect of fast-tracked school expansion on the quality and effectiveness of teaching-learning practices and environment.

On the other hand, this scenario tells us to project the future expansion demand for secondary and higher education. In line with this, it is concluded that the relevance of lower levels of education not only to train students so that they become fit for a successful university career but also to create a sufficiently large basis of capable students from which the ablest can eventually be selected for higher education, (Michaelowa and Bourdon, 2006). This shows that there are mediated connections from primary education to secondary education to tertiary education. Supporting the result stated in Table 1 though an increasing number of primary schools necessarily indicates an increased number of enrolments, principal respondents explained that still due to the existence of a significant number of pastoral and semi-pastoralist communities in the zones, full provision of the formal school system is becoming difficult.

Similarly, the researchers have observed that the expansion of primary schools seems to be at the expense of the quality of the school infrastructure and teaching-learning activities. In another word, the efforts made in order to address access to basic education target, and the physical expansion of schools did not give much emphasis to the quality of infrastructure, teachers’ qualification level, and students’ learning interests. In some primary schools (especially rural schools) quality of the schools has deteriorated, infrastructures are limited, shortage of qualified teachers (especially language teachers), and schools were technologically not supported.

### 3.1 Performance of the schools

Public primary school educational performance was measured mainly by gross enrolment rate and net intake rate in grade 1. Additionally, dropout rate, repetition rate, gender parity index, and ratios of qualified primary school teachers were used as performance indicators. The following Table 2 shows a summary of the average school-age population.

**Table 2:** School-age population of the zones.

Gender/Year	2017	2018	2019
Male	108241	110031	114590
Female	106082	108088	113911
Total	214323	217884	228499

As indicated in Table 2 above, eligibility for primary education was consecutively increasing from year to year. This shows that more primary school expansion needs to be done as a response to pupils’ age categories. A _X_^2^-test of independence result indicates that there is a statistically significant relationship between gender and the school-age population at _X_^2^ = 6.65, df = 2, and p-value = 0.00). More school-age boys were found outside the school from year to year due to different factors. The main economic activities of the rural districts are cash crop growing areas particularly coffee and agricultural activities are mainly practiced. It is assumed that boys have largely engaged in these economic activities to sustain their livelihood, earn quick incomes, and subsidize their families’ expenses. According to the discussion made with the discussants, large numbers of communities live in geographically dispersed areas which creates a challenge to expand formal and basic education to ensure equitable primary education access. In spite of increased enrolment rates in some districts and insignificantly increasing at another district to meet the ESDP V goals. There was also a significant variation in enrolment of male and female students. Table 3 below indicates the gross enrolment rate of students in the zones by gender expressed in percentage.

**Table 3:** Students gross enrolment rate (GER) of the schools in the zones expressed in percentage.

Gender/Academic year	2017	2018	2019
Male GER	134	120	131
Female GER	106	102	104
Average GER	120	111	118
Total	240	222	235

According to the plan, the gross enrolment rate target to be met at the end of the strategic year i.e.2020 was 103%. It is planned by assuming that it would be realized by increasing enrolment of students of the correct age for the correct grade level. However, as it is indicated in Table 3 above the present study indicates the average gross enrolment rate of the selected schools is 120% in 2017, 111% in 2018, and 118% in 2019 which is far from the planned target. This shows that children older than 14 years old are enrolled into primary schools due to the presence of lower access to primary education in some parts of the study areas (i.e. nomadic area) in which 7 years old is a national standard to join grade one. The result also indicates that relatively female students’ participation in primary school is better than that of male students. This is happened first due to the expansion of primary schools to the remote rural areas significantly reducing the distance to be traveled from home to school in which female students were benefited more than their male counterparts. Second, the raising of family awareness to send their daughters to school during the last two decades in comparison to the previous practices would contribute to getting a large number of female students’ enrolment into primary schools. A _X_^2^ test is calculated to examine if there was a statistically significant difference, but the result indicated that this observed difference between male and female enrolment rate is not statistically significant at x^2^ = 0.19, df = 2, and p-value = 0.911. Whereas according to the national education statistics annual abstract of 2017, the gross enrolment rate for primary schools was 111%. The following Table 4 reflects the net intake rate trends of primary schools in the study area expressed in percentage.

**Table 4:** Students net intake rate (NIR) trends of the school in the zones in percentage.

Gender/Academic Year	2017	2018	2019
Male NIR	93.42	106.29	104.86
Female NIR	111.58	92.94	122.51
Average NIR	102.5	99.92	113.68
Total (in %)	205	199.2	227.4

It is understandable that the age level at which first-grade enrolment is made determines the completion rate of education. In this study official entrance age to first grade is used to know the students’ entrance age of different years in the study area by considering 100% as standard. Accordingly, Table 4 above indicates the percentage of students who are enrolled to grade one at the correct age in different academic years. As indicated in Table 1 02.5 % in the 2017 year, 99.92 % in the 2018 year and 113.68 % in the 2019 year are recorded as the average net intake rate of Bench-sheko, Kafa, and Sheka zones in the three consecutive years. In comparison to the national achievements of the net intake rate performance which is 107.1% in 2017, 125. 3% in 2018 and 142.95% in 2019, the registered performance of the study areas was better. As the Chi-square test of independence showed the difference observed between male and female intake rates is not statistically significant at x^2^ = 3.08, df = 2, and p-value = 0.21. However, when it is compared with the net intake rate target which is 105 % for males and 101% for females, still some variations are observed that need to be reconsidered. For the fact that the result of the test of independence is statistically insignificant, the variation between the two sexes is decreased slightly from 18.2% in 2017 to 17.65 percentage points in 2019. The following Table 5 shows dropout rate trends of primary schools of the study area expressed in percentage.

**Table 5:** Students dropout rate trends of the school in the zones expressed in percentage.

Gender/Academic Year	2017	2018	2019
Male dropout rate	2.88	5.15	4.08
Female dropout rate	2.48	4.63	4.75
Average dropout rate	2.68	4.89	4.42
Total	5.36	9.78	8.83

Dropout and repetition rates are very important variables used to measure the efficiency and effectiveness of primary schools. In this study dropout rate of students is measured by students who left school between the beginning of each year. According to the table dropout rate of female students was significantly increasing from 2017 to 2019 academic years. However, the difference observed between male and female dropouts from year to year was statistically not significant at x^2^ = 0.12, df = 2, and p-value = 0.948. It was also observed that students do not drop out once and for all rather they return back to school and join education after one or more years. The result stated that many more activities are expected to be done by the schools since the percentage of dropout rate was higher than (i.e.4.42%) the target to be met. Besides, in Ethiopia rural children are sixty times more likely to drop out than urban children (Education for All global report, 2007).

From the study, the extent of the educational dropout was high in the academic years. In this regard, the school principals expressed their view that it is due to most of the students’ failure to study hard, lack of interest in education, interest to get income in short and low future success expectation leads them to fail in their achievement and then gradually to drop out. A study by Doll Jacob et al. (2013). stated, the specific dropout causes were categorized into push and pull factors in which the former includes school-consequence related to students’ misbehaviours whereas the latter factors are related to opportunities and incentives including jobs and family issues found outside of the schools. Likewise, this study also substantiates that all the factors were present for students’ dropout from the schools. The following Table 6 deals with primary schools’ repetition rate trends expressed in percentage.

**Table 6:** Students repetition rate trends of the school of the zones expressed in percentage.

Gender/Academic Year	2017	2018	2019
Male	2.53	4.98	4.64
Female	2.61	5.10	5.19
Average	2.57	5.04	4.92
Total	5.14	10.09	9.83

Repetition of the same grade was the other problem of the schools. This finding indicates a significant number of students have repeated the same grade at least for two or more years in which the students left schools at different times (i.e. at the earlier, at the midst, or later periods of academic years). As to Table 6, repetition rates in the schools were the highest in the 2018 and 2019 years which is 5.04% and 4.92% respectively. It was also observed that female students repeat the same grade more frequently than male students with an increasing rate. A chi-square test was conducted to assess whether female students were less or more repeating the same grade compared with male students. But, the observed difference was not statistically different at x^2^ = 0.01, df = 2 and p-value = 0.994.

Though the repetition rate of students of primary schools was increasing in the years indicated, it is slightly less than the national repetition rate which was 6.8 % for females and 7.5% for males according to educational statistics annual Education Statistics Annual Abstract, 2016 and very far from the target to be met which is 2%. Different educational scholars conclude that increased repetition rate of students could lead to inefficient education practices though the reason for the repetition varies from school to school. The following Table 7 is the summary of qualified teachers’ ratio trends of the primary schools used for the study purpose. Hence, as per the Education Sector Development Program V (ESDP V,2016–2020), a qualified teacher is a teacher who are qualified in teaching program training to teach at any level of education designed by the Ministry of Education and assigned to primary schools as per the qualification standard of the country (diploma holder for the first cycle i.e. 1 up to 4 grade and first-degree holder for the second cycle i.e. 5 up to 8 grades).

**Table 7:** Teacher-students’ ratio trends of the primary school of the zones.

Academic year	2017	2018	2019
Total enrolment	564623	589486	636787
Qualified teacher	9021	11298	13689
Teacher-students ratio	1: 63	1: 55	1: 50

According to the finding summarised in Table 7, teacher to student ratio was fallen from 1:63 in 2017, to 1:50 in 2019 years. The observed difference from year to year is statistically significant at x^2^ = 484.67, df = 2 and p-value = 0.00. In this case, compared with the national standard that is 1:50, the schools are performing to the standard. However, school principals have frequently raised about lack of experienced and qualified teachers which intern leads to a serious problem to handle teaching and learning activities, impacts the quality of education provided by the schools, limits education access opportunities and effectiveness of the schools. Besides, the discussants have especially justified that graduates of applied subjects were recruited to teach because experienced and qualified teachers were leaving the school. These kinds of teachers didn’t have any teaching skill training and they are not qualified teachers. The following table is the summary of the primary school gender parity index of the study area.

Gender parity (GPI) in primary education was used to measure the realization of the target stated in education sector development program V regarding access to quality education. Accordingly, this study confirmed that though a considerable effort was made by the schools to increase female students’ enrolment to primary schools as it is described in Table 8 above, gender parity index of the study area was decreasing. Hence, a Chi-square test of independence result showed that this difference observed from year to year is significant at x^2^ = 120.94, df = 2, and p-value = 0.00. The fact that commendable achievements were registered by the government in addressing the quest for access to universal education and enhancing equity by increasing female education participation, still the target is not met. The effect of gender inequality in lower grades on tertiary education outcomes is more serious for disadvantaged female students than their counterparts, (Michaelowa, 2007). Similarly, it is understandable that students’ economic background was a determining variable to the learning success of female students and when positively present it will enhance their active engagements. In addition to the economic background, geographically distant locations of their residence, political insecurity of the environment and physical health situations such as physically challenged female students were influencing them to actively participate in education.

**Table 8:** Gender parity index (GPI) trends of the primary school of the zones.

Gender/Academic Year	2017	2018	2019
Male	314279	329332	372584
Female	250344	260164	285633
GPI	0.80	0.79	0.77
Total	564,623	589,496	658,217

The study which was presented on the global education digest in 2010 concluded that globally, girls are more likely to never enter primary school than boys. This implies that more works need to be done at the international, national, and local level to achieve and create equity and reduce disparity. The researchers believe that realizing gender parity index in school is a manifestation of educational equity and creating equal opportunities which implies addressing education development targets of the nation. Besides, in a global sense, a research report on global female education participation disclosed that female students’ enrolment in primary education was rising faster than that of males and it helped to close the gender gap at the primary level (World Women’s, 2015).

Correspondingly, to maintain education equity it is assumed that primary schools have the responsibility to provide equal access for students with special need educations. In this regard based on the educational reports of the schools, the number of students with special need education admitted to school was increasing. However, it has been identified through observations that poor progress and practices of supporting children with special need education was in place. Special facilities supposed to be designed for this type of student are almost insignificant. School principals who have participated in focus group discussions substantiated that though their enrolment rate is increasing from year to year, challenges like lack of awareness by schools and the larger community about the academic, social, and economic support that supposed to be given for students with special need education was very less. Commitment to implement the nationally designed provisions and facilities to support them was weak. Poor school infrastructure facilities and weak pedagogical skill exposures of teachers were also identified as a challenge.

### 3.2 Financial strengths of the schools

School principals who have participated in the interviews concluded that though public funding of primary schools is increasing from time to time, compared to the enrolment rate its ratio remains relatively low. Primary schools have faced many challenges including high enrolment rate, low gender parity index, poor learners’ achievement, lack of staff incentives, and insufficient educational materials. Funding public primary education is one of the major responsibilities of the regional government. Regional government allocates very limited budgets through zonal and district education bureaus for the schools that is used for core activities such as maintenance, purchase of equipment and furniture, and building of new classrooms. The main budget is for the salaries of teachers and administrative staff. A small budget is allocated for non-salary activities.

Shortage, late approval of school budget, and irregularities of the allocated amount was a serious problem encountered by most primary schools of the study area and which apparently affects their daily planning and decision-making activities. In this case, late approval of the budget ranges for months, and sometimes it could be for a semester. Though the schools have a mandate to generate additional resources, very limited resources are generated for various reasons. Agricultural products are a source of internal income. However, a very small amount of budget is collected from agricultural products. Parents are found to be covering their students’ expenses (e.g. cost to buy uniform, school construction fees, registration fees). The insufficient budget allocated for primary schools negatively affects their performances. Additionally, budget shortage prevents the schools from paying incentives for teachers. The amount of resources collected from the community for different means varies due to different economic or earning capabilities of the community to contribute to the schools. This was peculiar to particularly rural schools that have weak economic potential and which suffer from insufficient budgets to implement their plans. The budget shortage is particularly challenging in rural schools where community contributions are small and students are exempted from paying school fees.

Mismanagement of school resources like farmland, crops, trees, and grasses was also reported as a problem. A school board and Parent Teacher Association (PTA) is responsible for the management, organization, and administration of the school budget in general. Not only budget shortage but also a decision for utilization is with full of challenges and time taking. School principals have no autonomy to decide on the budget and use it when the need arises. For instance, if the school want to buy accessories for daily activities like photocopy machine and computers, it is a must to request the school board or parent-teacher association’s consent.

### 3.3 Challenges of the schools

The discussants repeatedly said that primary schools have a problem with students’ learning motivation which was demonstrated by high dropout, high repetition rates, and low teachers’ teaching commitment. The schools have a shortage of experienced qualified teachers, a limited number of classrooms and sanitary facilities, and low support given to children with disabilities. However, gross enrolment rates were increasing from year to year relative to the performance of the school recorded during ESDP IV. According to the discussant’s explanation, though there was no culture of regularly rating student learning interest both at national as well as at regional education bureaus, the present practice and observation indicate that students’ learning interest is poor. These are manifested by the situation in which significant number of students are not motivated to actively engage in learning activities and take responsibility for their academic records. After they graduate from primary school a significant number of students show difficulty with numeracy and literacy skills. The schools’ yearly performance report attributes these problems to the socio-economic background of students’ parents and the nature of the economic activities of the environment that attracts the attention of the students more than the school. The discussants from the schools also affirmed that weak schools’ budget utilization and less support from the community in planning, decision-making, and resource mobilization were mentioned as major challenges influencing their performances.

Additionally, it is explained that the present earning salary and incentive practice of primary school teachers is very poor and does not sufficiently cover their basic expenses which in turn resulted in a lack of commitment to teaching, supporting students and even leads to high teachers’ turnover year in year out. This by itself caused qualified and experienced teachers to even leave schools very shortly and their engagement in professional development activities was also weak or not at all. In this regard, the respondents in group discussion expressed their opinion by saying a systemic, integrated, and transparent performance evaluation mechanism of primary school teachers and associated benefits are still missing which leads to less commitment to engage in continuous professional development activities.

## 4 Conclusions

It is found that the schools were progressing in access. However, much more work is expected to be done by the School Leaders, Non-Governmental Organizations (NGOs), Partners, Parents, Local, Regional and National Governments to meet the nationally expected targets of access to primary education without compromising its quality. Enrolment rate was increasing with the existing infrastructural facilities. This situation was creating burdens on the school leadership, teachers teaching load and effectiveness, and students learning interests. Qualified experienced teachers are leaving the schools and joining different careers to get extra income in order to subsidize their living costs. These problems lead the school in order not to progress in line with the expectations and affect the quality of teaching-learning activities. The researchers recommend that to significantly reduce this problem intervention is needed at the policy level which may include making inclusive policy reform, critical implementation, monitoring, and evaluation regarding standardizing the number of students that each school should enrol, quality and quantities of teachers needed, minimum school facilities required, standardized and diversified funding system and the overall teaching and learning packages of primary schools. Creating shared responsibility among the school leadership, local administrators and the community is a key to systematically avert problems and enhancing educational efficiency by reducing dropout and repetition rates.

## Declarations

### Author contribution statement

Miressa Yadessa: Conceived and designed the experiments; Performed the experiments; Analyzed and interpreted the data; Wrote the paper.

Matheas Shemelis: Analyzed and interpreted the data; Contributed reagents, materials, analysis tools or data; Wrote the paper.

### Funding statement

This research did not receive any specific grant from funding agencies in the public, commercial, or not-for-profit sectors. This work was supported by Mizan-Tepi University.

### Data availability statement

Data will be made available on request.

### Declaration of interests statement

The authors declare no conflict of interest.

### Additional information

No additional information is available for this paper.

## Appendix A Supplementary data

Questionnaires Questionnaires
