# Supplementary material for: In-depth analysis of primary schools performance, financial strength and challenges in Bench-Sheko, Kafa and Sheka Zones, Ethiopia
Source: Heliyon. 2022 Mar 16;8(3):e09116. doi: 10.1016/j.heliyon.2022.e09116 (PMC9280377; doi:10.1016/j.heliyon.2022.e09116)
Supplement: Questionnaires [file mmc1.docx]

**Mizan_Tepi University**

**A questionnaire to be filled Schools Principals /Unit leaders and or Teachers**

The purpose of this questionnaire is to obtain information about the performance and financial strength of public primary schools of Benchi Maji, Kaffa and Sheka Zone. The study aims to generate valid and empirically based statements in line with ESDP V standards. Your real and genuine responses to each item will have a pivotal role for accuracy and reliability of the research. Therefore, you are kindly requested to go through all questions and fill appropriately based on your school’s yearly performances. Your response will be used only for this study and its confidentiality is safeguarded. Your participation in this study is absolutely based on interest and your freedom to decline the participation is maintained. Don’t write your name.

**PARTI: BACKGROUND INFORMATION**

**Direction I:** The following questions are aimed to get general data about your background information and your school profile. Please read the statements carefully & give appropriate response for each question.

1. Zone: ____________________________________
2. Woreda: __________________________________
3. School Name: ______________________________
4. School level: ______________________________
5. Your role in the school: ______________________
6. Experience: _______________________________
7. Sex: _____________________________________

**Direction II:** Table below indicates key access performance indicators of primary schools as per ESDP V that related to primary education programs. Look at the items carefully and fill the question appropriately.

|  | **Items / Indicators** | **2017** | **2018** | **2019** |
| --- | --- | --- | --- | --- |
| 1 | Grade 1 NIR female |  |  |  |
| 2 | Grade 1 NIR male |  |  |  |
| 3 | Grade 1–4 GER female |  |  |  |
| 4 | Grade 1–4 GER male |  |  |  |
| 5 | Grade 1–4 NER female |  |  |  |
| 6 | Grade 1–4 NER male |  |  |  |
| 7 | Grade 5–8 GER female |  |  |  |
| 8 | Grade 5–8 GER male |  |  |  |
| 9 | Grade 5–8 NER female |  |  |  |
| 10 | Grade 5–8 NER female |  |  |  |
| 11 | Grade 1–8 GER female |  |  |  |
| 12 | Grade 1–8 GER male |  |  |  |
| 13 | Grade 1–8 NER female |  |  |  |
| 14 | Grade 1–8 NER male |  |  |  |

GER: gross enrollment rate, NER: Net enrollment rate, NIT: Net intake rate

15. How do you evaluate enrolment and educational infrastructure provision practices of your school? Discuss briefly.

**Direction II**: The following items are used to measure status of educational efficiency of your school. Fill the information of your school carefully as per the request.

| **No** | **Items / Indicators** | **2017** | **2018** | **2019** |
| --- | --- | --- | --- | --- |
| 1 | Grade 1 dropout rate female |  |  |  |
| 2 | Grade 1 dropout rate male |  |  |  |
| 3 | Grade 1–8 dropout rate female |  |  |  |
| 4 | Grade 1–8 dropout rate male |  |  |  |
| 5 | Grade 1–8 repetition rate female |  |  |  |
| 6 | Grade 1–8 repetition rate male |  |  |  |
| 7 | Completion rate to Grade 8 female |  |  |  |
| 8 | Completion rate to Grade 8 male |  |  |  |

9. Dear respondents, what does the situation of dropout and repetition rate practice of your school looks like? What do you think are the reason for the problems? Discuss

**Direction III**: The following items are used to indicate qualification of teachers. Fill the information of your school carefully as per the request.

| **No** | **Items / Indicators** | **Certificate** | **Diploma** | **Degree** | **Masters** |
| --- | --- | --- | --- | --- | --- |
| 1 | Grades 1–4 teachers appropriately qualified female |  |  |  |  |
| 2 | Grades 1–4 teachers appropriately qualified male |  |  |  |  |
| 3 | Teachers in Grades 1–8 that are licensed female |  |  |  |  |
| 4 | Teachers in Grades 1–8 that are licensed male |  |  |  |  |

5. What do you think the appropriateness of the qualification of teachers teaching in your school? What constraints did you observe in this regard throughout your teaching experience? What solutions would you suggest to overcome the constraints? Discuss.

**Direction IV:** The following items are used to examine the level of equity in your school. Fill the information of your school carefully as per the request.

| **No** | **Items / Indicators** | **2017** | **2018** | **2019** |
| --- | --- | --- | --- | --- |
| 1 | GPI in Grades 1–8 (index) |  |  |  |
| 2 | Enrolment rate of children with SNE, Grades 1–8 |  |  |  |
| 3 | Females share of school principals and supervisor |  |  |  |

4. How do you evaluate the educational participation of female students in general and children with special need in particular? What challenges did you observe in this regard throughout different years? What solutions would you suggest to overcome the constraints? Discuss.

11. Discuss about the financial source of your school, utilization practices and challenges? What solutions would you suggest to overcome the challenges?

**THANK YOU FOR YOUR COOPERATION!**
